# Genetically predicted C-reactive protein mediates the association between rheumatoid arthritis and atlantoaxial subluxation

**DOI:** 10.3389/fendo.2022.1054206

**Published:** 2022-12-16

**Authors:** Jiaqin Yuan, Xiaoqin Xiong, Bin Zhang, Qingyuan Feng, Jinglin Zhang, Wenting Wang, Jia Tang

**Affiliations:** ^1^ Department of Orthopedics, The Second People’s Hospital of Yibin, Yibin, Sichuan, China; ^2^ Department of Orthopedics, Yibin Hospital, West China Hospital of Sichuan University, Yibin, Sichuan, China; ^3^ Department of Pediatrics, The Affiliated Hospital of Southwest Medical University, Luzhou, China; ^4^ Rheumatism Immunity Branch, Weifang People’s Hospital, Weifang, Shandong, China; ^5^ Wuxi School of Medicine, Jiangnan University, Wuxi, Jiangsu, China; ^6^ Department of Occupational Disease, Yibin Center for Disease Control and Prevention, Yibin, Sichuan, China; ^7^ Department of Anesthesiology, The Second Affiliated Hospital of Hainan Medical University, Haikou, China; ^8^ Department of Pediatrics, Daping Hospital, Army Medical University, Chongqing, China

**Keywords:** Mendelian randomization, rheumatoid arthritis, C-reactive protein, atlantoaxial subluxation, upper cervical instability

## Abstract

**Objective:**

Investigating the causal relationship between rheumatoid arthritis (RA) and atlantoaxial subluxation (AAS) and identifying and quantifying the role of C-reactive protein (CRP) as a potential mediator.

**Methods:**

Using summary-level data from a genome-wide association study (GWAS), a two-sample Mendelian randomization (MR) analysis of genetically predicted rheumatoid arthritis (14,361 cases, and 43,923 controls) and AAS (141 cases, 227,388 controls) was performed. Furthermore, we used two-step MR to quantitate the proportion of the effect of c-reactive protein-mediated RA on AAS.

**Results:**

MR analysis identified higher genetically predicted rheumatoid arthritis (primary MR analysis odds ratio (OR) 0.61/SD increase, 95% confidence interval (CI) 1.36-1.90) increased risk of AAS. There was no strong evidence that genetically predicted AAS had an effect on rheumatoid arthritis risk (OR 1.001, 95% CI 0.97-1.03). The proportion of genetically predicted rheumatoid arthritis mediated by C-reactive protein was 3.7% (95%CI 0.1%−7.3%).

**Conclusion:**

In conclusion, our study identified a causal relationship between RA and AAS, with a small proportion of the effect mediated by CRP, but a majority of the effect of RA on AAS remains unclear. Further research is needed on additional risk factors as potential mediators. In clinical practice, lesions of the upper cervical spine in RA patients need to be given more attention.

## Introduction

Rheumatoid arthritis (RA) is a chronic inflammatory immune system disease characterized by synovitis and cartilage destruction, which mainly affects the synovial membrane, tendon sheaths and synovial bursae of the joints (1). It mainly manifests as clinical symptoms such as joint pain, stiffness, swelling, deformity, and dysfunction (2). Its global prevalence is approximately 1% and ranks 42nd among disabling diseases worldwide (3). As the global population ages, the prevalence continues to increase. Due to RA’s high mortality and morbidity rates, patients’ quality of life is lower, and the economic burden on society is greater. The National Audit Office (NAO) reports that RA costs the UK approximately £560 million a year in health care costs, not including the cost of sick leave and work-related disability (4).

The active segment of the cervical vertebra is the basic functional unit of the cervical spine. It consists of two adjacent cervical vertebrae and their attached soft tissues and is the smallest functional unit of the cervical vertebra. Cervical instability refers to excessive or abnormal cervical spine movement that cannot maintain the normal position between the vertebral bodies under physiological loads (5). Atlantoaxial subluxation (AAS) in RA patients mostly involves the atlantoaxial joint, which may be caused by head and neck trauma, congenital diseases (bone dysplasia), autoimmune diseases (rheumatoid arthritis), etc. However, the exact reason is not yet clear. Observational studies have shown that upper cervical instability occurs in 29.6% of RA patients, of which atlantoaxial subluxation accounts for 24.6% (6). However, epidemiological studies may suffer from measurement error, uncontrolled confounding factors, and reverse causality. Ultimately, the results may be subject to various biases. Therefore, a design is needed to avoid or reduce some biases further to demonstrate the causal relationship between RA and AAS.

Moreover, potential pathways related to RA and AAS have not been investigated. Previous studies have provided evidence that C-reactive protein (CRP) is elevated in both RA and AAS (7, 8). Consequently, CRP might be a potential mediator between RA and AAS.

Mendelian randomization (MR) is a potential causal inference method that uses genetic variation as an instrumental variable to obtain the effect of exposure factors on outcomes from observational data (9). MR can reduce the effects of nonmeasurement errors or confounding factors while avoiding reverse causality through Mendelian inheritance laws (9). Therefore, we aimed to (i) determine whether RA is causally related to AAS and (ii) assess the extent to which CRP mediates the effects of RA on AAS.

## Methods

### Study design

The data used in our analysis were publicly available and were approved by the institutional review committee in the respective studies. Therefore, no further sanctions were needed. All generated results are presented in the article and its supplements.

In this study, we explored the reciprocal causal relationship between rheumatoid arthritis and atlantoaxial subluxation by two-sample, bidirectional mendelian randomization. In our study, single nucleotide polymorphisms (SNPs) were defined as instrumental variables (IVs) (10).

### GWAS summary data sources

The data used in our study were all publicly available, and the participants in the GWAS were of European ancestry. The genetic associations of RA were derived from a GWAS meta-analysis by Ha and colleagues (11), which included 14,361 RA case and 43,923 controls. All cases met the 1987 American College of Rheumatology criteria or were diagnosed as RA by a rheumatologist. ninety-one percent of individuals were serologically positive for anti-CCP antibodies or rheumatoid factor. Additional details are shown in **Supplementary Table S1**.

Data on AAS were drawn from the GWAS summary data sources on the FennGenn consortium, which is available at https://www.finngen.fi/en (AAS including 141 cases and 227,388 participants) (12). Individuals with ICD codes [ICD-10 M43.3 “Recurrent atlantoaxial subluxation with myelopathy” and ICD-10 M43.4 “Other recurrent atlantoaxial subluxation”] were characterized as AAS cases.

Summary statistics on CRP levels were obtained from a published GWAS meta-analysis that included 78 studies of European ancestry, with the largest sample size thus far (sample size  = 204,402) (13). The study design, such as sample collection, quality control procedures, and imputation methods, were described in the original publication. Additional details are shown in **Supplementary Table S2**. All GWAS data are from different consortia or organizations, and thus there is no sample overlap.

### Instrumental variable selection and data harmonization

We included SNPs that were genome-wide significant (P < 5 × 10^−8^). If there were no significant genome-wide SNPs as IVs, SNPs with less than a genome-wide significance level (P < 5 × 10^-6^) were used as candidate IVs. Then, these SNPs were clustered based on linkage disequilibrium (window size = 10,000 kb and r^2^ < 0.001). Estimated levels of linkage disequilibrium from the 1000 Genomes Project based on European samples (14). If a particular exposed SNP was not present in the outcome dataset, proxy SNPs were used by LD tagging. Palindromic and ambiguous SNPs were excluded from IVs for Mendelian randomization analysis (15). The F statistic was calculated by the variance explained by SNPs for each exposure, i.e. [(N – K – 1)/K]/[R^2^/(1 – R^2^)], where K is the number of genetic variants, N is the sample size. We removed weak instrumental variables (F-statistics < 10) (16, 17).

### Statistical analysis

We performed MR analysis using R software (version 4.2.0, http://www.r-project.org) and the “Two-Sample MR” package (version 0.5.6) (18). MR-Pleiotropy RESidual Sum and Outlier (MR-PRESSO) and robust adjusted profile score (MR.RAPS) were performed using the R packages “MRPRESSO” and “MR.raps”, respectively. Calculation of statistical power for Mendelian randomization was performed using mRnd (https://cnsgenomics.shinyapps.io/mRnd/). And we applied a PhenoScanner search to assess all known phenotypes related to the considered genetic instruments in our analyses.

### Primary analysis

**Figure 1:**
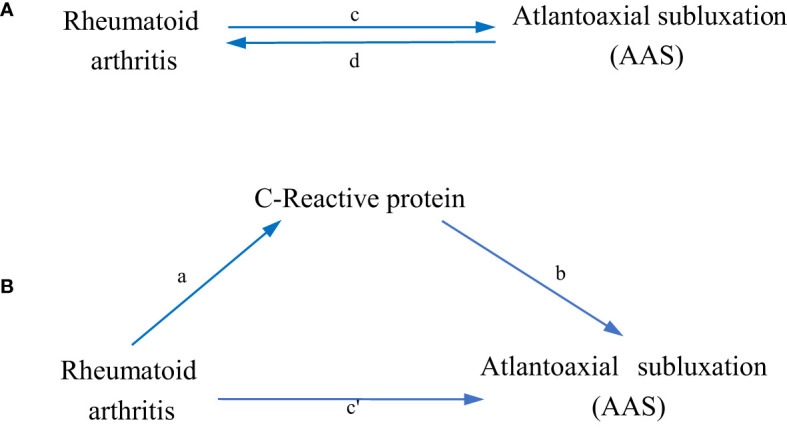
Diagrams illustrating associations examined in this study. **(A)** The total effect between rheumatoid arthritis(RA) and atlantoaxial subluxation(AAS). *c* is the total effect using genetically predicted RA as exposure and AAS as outcome. *d* is the total effect using genetically predicted AAS as exposure and RA as outcome. **(B)** The total effect was decomposed into: (i) indirect effect using a two-step approach (where *a* is the total effect of RA on CRP, and *b* is the effect of CRP on AAS) and the product method (*a *× *b*) and (ii) direct effect (*c′* = *c* – *a* × *b*). Proportion mediated was the indirect effect divided by the total effect.

**Figure 1** shows a schematic summary of the analysis. We conducted a two-sample bidirectional MR to evaluate the mutual causality between RA and AAS (**Figure 1A**), which was designated as the total effect.

Inverse variance weighting (IVW) uses meta-analysis to combine the Wald ratios of causal effects for each SNP (15, 19). Then, MR-Egger (20) and weighted-median (21) methods were used as a complement to IVW. Different methods adapted to different validity assumptions were applied to obtain MR estimates. The application of IVW is based on the premise that all SNPs are valid instrumental variables. Therefore, this method can obtain accurate estimation results. MR-Egger assesses directional pleiotropy for instrumental variables, where the intercept can be interpreted as an estimate of the average pleiotropy of genetic variation. The weighted median has the advantage of maintaining higher precision (smaller standard deviation) compared to the MR-Egger analysis. In the presence of horizontal pleiotropy, the weighted median provides a consistent estimate even if 50% of the genetic variants are invalid IVs (22).

### Mediation analysis

**Figure 2:**
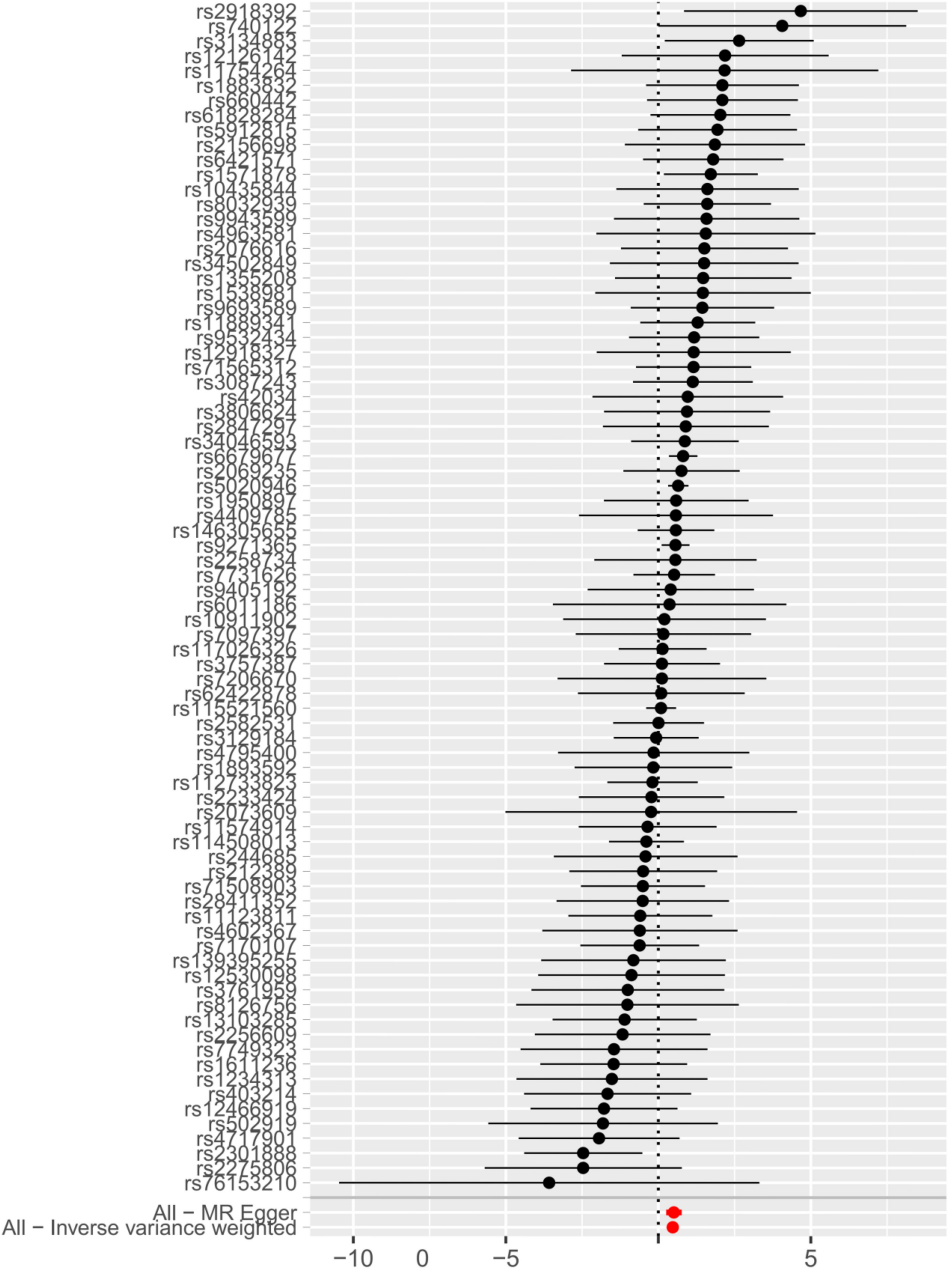
Forest plot to visualize causal effect of each single SNP on total AAS risk.

We further performed a mediation analysis using a two-step MR design to explore whether CRP mediates the causal pathway from RA to AAS outcome (**Figure 1B**). The overall effect can be decomposed into an indirect effect (through mediators) and a direct effect (without mediators) effect (23). The total effect of RA on AAS was decomposed into 1) direct effects of RA on AAS (*c’* in **Figure 1B**) and 2) indirect effects mediated by RA through the mediator (*a × b* in **Figure 1B**). We calculated the percentage mediated by the mediating effect by dividing the indirect effect by the total effect. Meanwhile, 95% confidence intervals were calculated with the delta method (24).

### Sensitivity analysis

The causal direction of each extracted SNP to exposure and outcome was tested by using MR Steiger filtering (25). This method calculates the variance explained in exposure and results from the instrumental SNPs and tests whether the variance in the results is less than the exposure. “TRUE” MR Steiger results indicate causality in the expected direction, while “FALSE” results indicate causality in the opposite direction. We excluded SNPs with ‘FALSE’ results, indicating that it showed evidence of a major effect on the outcome rather than exposure.

Heterogeneity between SNPs was assessed using Cochran’s Q statistic and funnel plots (26, 27). Horizontal pleiotropy was detected using the MR-Egger intercept (20) method and the MR-PRESSO (28) method. If outliers were detected, they were removed, and we re-evaluated the MR causal estimates. If heterogeneity remained high after removal, the stability of the results was assessed using a random effects model, which is less susceptible to weaker SNP exposure associations. Finally, leave-one-out analysis was used to validate the effect of each SNP on the overall causal estimates.

## Results

### Association of RA with AAS

After removing palindromic and ambiguous SNPs, SNPs without proxy and SNPs with wrong causal directions identified by MR Steiger filtering, there were 80 SNPs in RA and 4 SNPs in AAS as instrumental variables (**Supplementary Tables S3, S4**). Since AAS did not reach the level of gene-wide significance for SNPs, SNPs with less than genome-wide significance (P < 5 × 10^-6^) were used as instrumental variables. The variance explained by and the F-statistic for SNPs instrumenting RA exposure were 5.8% and 45, respectively. Our study provides 100% power to detect the causal effect of RA on AAS risk.

**Figure 3:**
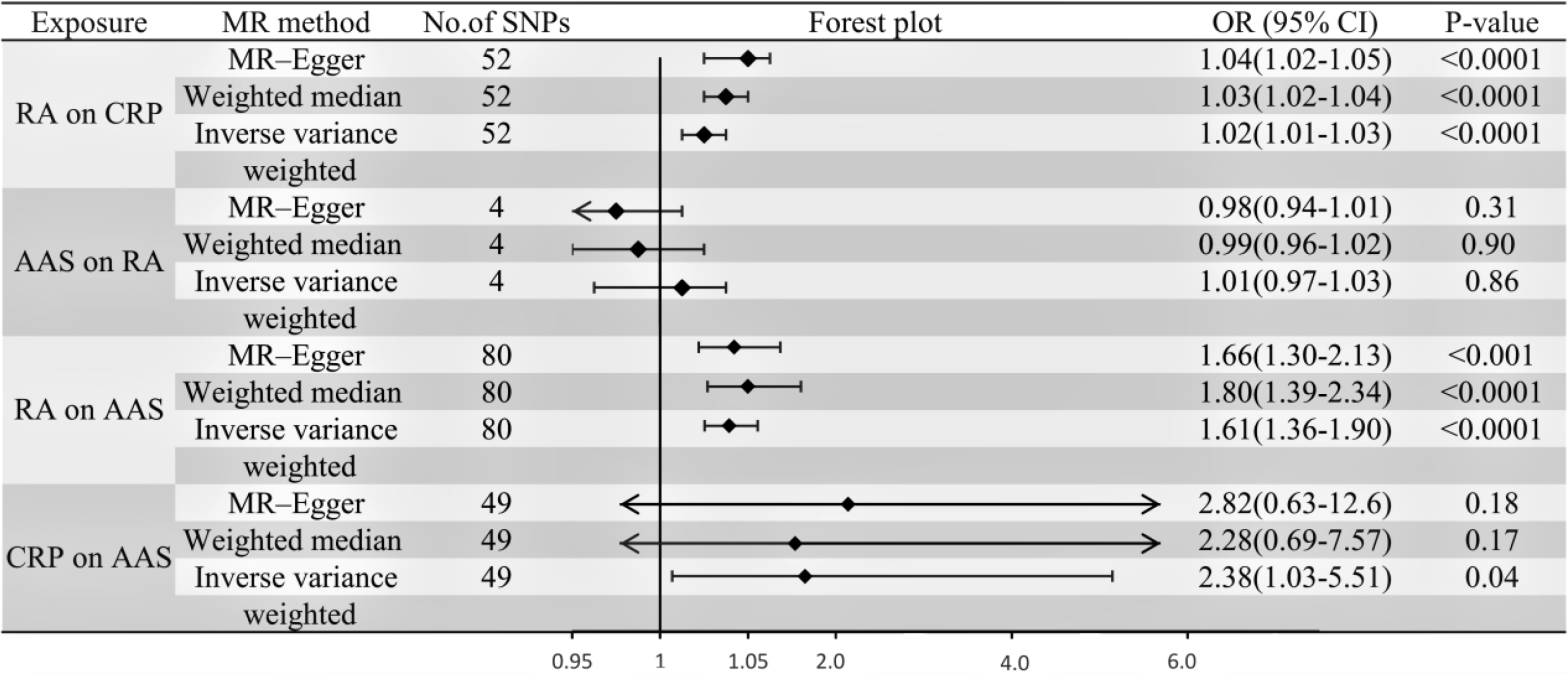
Forest plot to visualize the causal effects of CRP with RA and AAS.

IVW, MR-Egger, and weighted median regression were used to estimate the causal relationship between genetically predicted RA and AAS (**Figures 2**, **3**). Across all three MR methods, there was broad and consistent support for the positive association of RA with AAS (IVW odds ratio [OR] per SD increase in RA = 1.61 [95% CI, 1.36-1.90], P < 0.0001; MR-Egger OR per SD increase in RA = 1.66 [95% CI, 1.30-2.13], P < 0.001; weighted median OR per SD increase in RA = 1.80 [95% CI, 1.39-2.34], P < 0.0001). However, the results of our MR analysis showed no reverse causality for genetically predicted RA on AAS (i.e., no causality for genetically predicted AAS on RA.). The OR was 1.001 [95% CI, 0.97-1.03; p = 0.87] by using the IVW method. The results are shown in **Figure 3**.

### Association of RA with CRP

We extracted a total of 52 genome-wide significant SNPs as instrumental variables after removing palindromic and ambiguous SNPs, SNPs without proxies, and SNPs in the wrong causal direction identified by MR Steiger filtering (**Supplementary Table S5**). The variance explained by and F-statistic for SNPs instrumenting RA exposure were 3.3% and 39, respectively.

According to the IVW, MR–Egger and weighted median methods, genetically predicted RA was found to be positively associated with CRP risk (IVW method, OR, 1.02; [95% CI, 1.01-1.03], P<0.0001; MR-Egger method, OR, 1.04; [95% CI, 1.02-1.05], P<0.0001; weighted median method, OR, 1.03; [95% CI, 1.02-1.04], P<0.0001). The results are shown in **Figure 3**.

### Association of CRP with AAS

Genetic instruments for CRP explained 1.3% of its variance, with an F-statistic of 54. As shown in **Supplementary Table S6**, we presented all genetic instruments associated with CRP at the genome-wide significance level (P < 5 x 10^-8^). As shown in **Figure 3**, genetically predicted CRP was significantly positively correlated with AAS [OR=2.38, 95% CI, 1.03-5.51; P=0.04] by using the IVW method. The estimation directions of these three methods, IVW, MR-Egger and weighted median, were consistent.

### Proportion of the association between RA and AAS mediated by CRP

**Figure 4:**
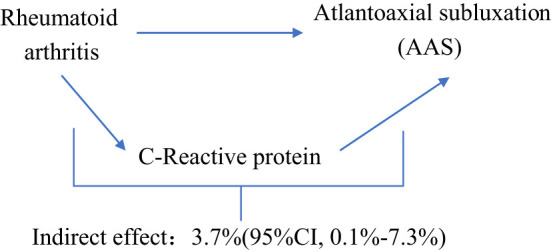
Schematic diagram of the CRP mediation effect.

We analyzed CRP as a mediator of the pathway from RA to AAS. We found that RA was associated with increased CRP, which in turn was associated with an increased risk of AAS. As shown in **Figure 4**, our study showed that CRP accounted for 3.7% of the increased risk of AAS associated with RA (proportion mediated: 3.7%; 95% CI = 0.1%−7.3%).

### Sensitivity analysis

Several sensitivity analyses were used to examine and correct for the presence of pleiotropy in causal estimates. Cochran’s Q-test and funnel plot showed no evidence of heterogeneity and asymmetry between these SNPs in the causal relationship between these SNPs (**Supplementary Table S7** and **Supplementary Figure S1**). In our study, the MR-Egger intercept showed weak evidence of pleiotropy at the directional level of the RA instrument (OR=0.996; 95% CI, 0.994-0.998; P=0.0001)), although no other MR-Egger intercepts did (**Supplementary Table S7**). We did not detect potential horizontal pleiotropy by using the MR-PRESSO global test (**Supplementary Table S7**). The effect of each SNP on the overall causal estimates was verified by leave-one-out analysis (**Supplementary Figure S2**). After removing each SNP, we systematically performed the MR analysis again for the remaining SNPs. The results remained consistent, indicating that all SNPs were calculated to make the causal relationship significant.

## Discussion

Recent studies (6, 29, 30) have examined the relationship between RA and AAS. However, the current evidence is limited to observational studies, and the results may be influenced by confounding factors. Our study aimed to illustrate the causal effects between RA and AAS. We used MR analysis to investigate the association between RA and AAS based on existing GWAS and to demonstrate whether the causal relationship between them is mediated through CRP. Our results suggested that genetically predicted RA was associated with an increased risk of AAS (61% increased risk of AAS for every 1 SD increase in RA), and 3.7% of this effect was mediated through CRP.

To date, we are the first to investigate the causal relationship between RA and the risk of instability in the upper cervical spine by MR methods, while also demonstrating CRP as their mediator. Our findings are consistent with previous findings from traditional observational designs. Yurube et al. (31) showed that atlantoaxial instability occurred in 43.6% of RA patients, with atlantoaxial subluxation being the most common (at least 32.1%) through a prospective follow-up study of RA patients with no initial cervical involvement for at least 5 years. Similarly, in a retrospective study of 240 RA patients, Kotecki et al. (32) showed that the incidence of cervical spine involvement in RA patients was 75%, the most common lesion was anterior atlantoaxial subluxation (approximately 58%), and C-reactive protein levels increased (OR, 19.0; 95% CI, 7.0–32.0; P = 0.016). However, both studies were of observational design. First, they had low response rates between the two groups, and second, their results were more influenced by reverse causality or other potential mixed effects than MR analyses.

Atlantoaxial instability refers to the structural changes and dysfunction of the atlanto-occipital joint and atlantoaxial joint between the atlas, the axis and the base of the skull due to various reasons (e.g., deformity, trauma, degeneration, tumor and infection), which lead to excessive or abnormal activity or abnormal position under physiological load. Atlantoaxial joint instability and dislocation are rare in normal adults and are mostly secondary to trauma and disease. Cervical instability can be further divided into atlantoaxial subluxation (AAS), vertical subluxation (VS) and subaxial subluxation (SAS). AAS is the most common, followed by VS, and SAS is less common (6). VS usually occurs after AAS. VS is considered a serious condition in RA patients because it can lead to sudden death (33). Synovitis is the initial link of rheumatoid arthritis, and it is also the basic pathological change. The characteristics of multiple synovial sacs of the atlantoaxial joint provide conditions for its involvement. At the same time, synovial tissue macrophages produce tumor necrosis factor to promote the inflammatory response of the atlantoaxial joint (34). Sorimachi et al. (35) have suggested that synovitis invades the atlantoaxial joint in three stages. First, the medial and lateral atlantoaxial joints are invaded, the joint capsule is destroyed, and the joint capsule is swollen and exuded; then, the synovium begins to proliferate, and the ligaments are edematous and destroyed, after which finally, it erodes hyaline cartilage and penetrates into subchondral bone to produce bone tissue destruction. In addition, the stability of the atlantoaxial joint mainly depends on the maintenance of the transverse ligament and other ligaments, which are characterized by high stiffness and insufficient willfulness. Another characteristic product of RA is pannus, which not only blocks the bone from obtaining nutrition through the synovium but also grows to the cartilage surface in the joint cavity, produces adhesions, and locally releases more inflammatory factors, proteolytic enzymes, etc. (36). When inflammation involves the transverse ligament, it not only destroys the fibrous structure and relaxes the ligament but also erodes the odontoid process and causes erosion and rupture near the attachment point, which finally leads to the instability of the atlantoaxial joint (37).

CRP, a member of the pentraxin family of proteins, consists of five 23 kDa subunits that can be increased 1,000-fold or more during infection, inflammation and tissue damage. Although hepatocytes are the main source of CRP, other cells, such as monocytes and lymphocytes, also produce CRP (38). Fang et al. (39) suggested that synovial tissue from RA patients also produces CRP. Therefore, one of the reasons for the increased CRP concentrations in synovial fluid and serum CRP levels in RA patients may be the local production of CRP in inflammatory synovial tissue (40). On the one hand, the interaction of CRP with Fcγ receptor I and FcγRIIA promotes the production of proinflammatory cytokines, leading to an amplification loop of the inflammatory response; on the other hand, CRP initiates bone destruction by inducing the receptor activator of nuclear factor-κB ligand protein and directly stimulating osteoclast generation, thus causing a vicious cycle between inflammation and bone destruction in RA (41). Therefore, CRP contributes to atlantoaxial joint instability by mediating synovial inflammation and bone destruction in RA.

Our findings also suggest that RA may increase the risk of atlantoaxial subluxation through other important mediators. Zhang et al. (6) showed that low hemoglobin levels may be associated with atlantoaxial instability in RA. This may be because low hemoglobin levels are partly a chronic inflammatory manifestation of the disease and are thought to be associated with joint damage in RA (42), thus showing a correlation with cervical instability. In addition, CD5^+^ B cells in RA patients can produce IgG with the help of T lymphocytes, and rheumatoid factor and IgG form immune complexes deposited in the synovium, which are blocked during the clearance process, resulting in bone destruction and fusion.

This study has several limitations. First, our analysis was performed using the European population, which limits its prevalence (43). Second, the smaller number of cases in AAS is in the GWAS dataset of AAS, and it is hoped that larger GWAS data will be available for validation in the future. Third, even if we took steps to identify and eliminate outlier variants, we cannot exclude the possibility that horizontal pleiotropy influenced our results. Fourth, we used summary-level statistics in our study, not individual-level data. Therefore, we cannot further explore causal links between subgroups such as females and males. Fifth, our study demonstrates that genetic prediction of rheumatoid arthritis mediated by C-reactive protein is 3.7%, which is very low. Thus, more studies are needed to quantify other mediators.

## Conclusion

In conclusion, our study identified a causal relationship between RA and atlantoaxial subluxation, with a small proportion of the effect mediated by CRP, but a majority of the effect of RA on atlantoaxial subluxation remains unclear. Further research is needed on additional risk factors as potential mediators. In clinical practice, lesions of the upper cervical spine in RA patients need to be given more attention.

## Data availability statement

The original contributions presented in the study are included in the article/**Supplementary Material**. Further inquiries can be directed to the corresponding authors.

## Author contributions

All authors designed this study. JT, WW, and XX performed the catalog and literature search and data extraction with suggestions and help from JY. JY, JZ, and XX performed the statistical analyses. All authors contributed to the data interpretation and manuscript writing. All authors contributed to the article and approved the submitted version.

## References

[1] Smolen JS Aletaha D McInnes IB. Rheumatoid arthritis. Lancet (London England) (2016) 388(10055):2023–38. doi: 10.1016/S0140-6736(16)30173-8 27156434

[2] Sharif K Sharif A Jumah F Oskouian R Tubbs RS. Rheumatoid arthritis in review: Clinical, anatomical, cellular and molecular points of view. Clin Anat (New York NY) (2018) 31(2):216–23. doi: 10.1002/ca.22980 28833647

[3] Cross M Smith E Hoy D Carmona L Wolfe F Vos T. The global burden of rheumatoid arthritis: estimates from the global burden of disease 2010 study. Ann Rheum Dis (2014) 73(7):1316–22. doi: 10.1136/annrheumdis-2013-204627 24550173

[4] Fazal SA Khan M Nishi SE Alam F Zarin N Bari MT. A clinical update and global economic burden of rheumatoid arthritis. Endocr Metab Immune Disord Drug Targets (2018) 18(2):98–109. doi: 10.2174/1871530317666171114122417 29141572

[5] Ng HW Teo EC Lee KK Qiu TX. Finite element analysis of cervical spinal instability under physiologic loading. J Spinal Disord Tech (2003) 16(1):55–65. doi: 10.1097/00024720-200302000-00010 12571486

[6] Zhang L Hu XH Wang QW Cai YM Zhao JX Liu XY. Population distribution and clinical characteristics in rheumatoid arthritis patients with cervical spine instability. Beijing Da Xue Xue Bao Yi Xue Ban (2020) 52(6):1034–9. doi: 10.19723/j.issn.1671-167X.2020.06.008 PMC7745262 33331310

[7] Magarelli N Simone F Amelia R Leone A Bosello S D'Antona G. MR imaging of atlantoaxial joint in early rheumatoid arthritis. Radiol Med (2010) 115(7):1111–20. doi: 10.1007/s11547-010-0574-4 20680496

[8] Ahn JK Hwang JW Oh JM Lee J Lee YS Jeon CH. Risk factors for development and progression of atlantoaxial subluxation in Korean patients with rheumatoid arthritis. Rheumatol Int (2011) 31(10):1363–8. doi: 10.1007/s00296-010-1437-y 20422194

[9] Emdin CA Khera AV Kathiresan S. Mendelian randomization. JAMA (2017) 318(19):1925–6. doi: 10.1001/jama.2017.17219 29164242

[10] Davey Smith G Hemani G. Mendelian randomization: genetic anchors for causal inference in epidemiological studies. Hum Mol Genet (2014) 23(R1):R89–98. doi: 10.1093/hmg/ddu328 PMC4170722 25064373

[11] Ha E Bae SC Kim K. Large-Scale meta-analysis across East Asian and European populations updated genetic architecture and variant-driven biology of rheumatoid arthritis, identifying 11 novel susceptibility loci. Ann Rheum Dis (2021) 80(5):558–65. doi: 10.1136/annrheumdis-2020-219065 PMC8053349 33310728

[12] Kurki MI Karjalainen J Palta P Sipilä TP Kristiansson K Donner K. FinnGen: Unique genetic insights from combining isolated population and national health register data. (2022) 2022:. doi: 10.1101/2022.03.03.22271360

[13] Ligthart S Vaez A Võsa U Stathopoulou MG de Vries PS Prins BP. Genome analyses of >200,000 individuals identify 58 loci for chronic inflammation and highlight pathways that link inflammation and complex disorders. Am J Hum Genet (2018) 103(5):691–706. doi: 10.1016/j.ajhg.2018.09.009 30388399 PMC6218410

[14] 1000 Genomes Project Consortium Abecasis GR Altshuler D Auton A Brooks LD Durbin RM. A map of human genome variation from population-scale sequencing. Nature (2010) 467(7319):1061–73. doi: 10.1038/nature09534 PMC3042601 20981092

[15] Hemani G Zheng J Elsworth B Wade KH Haberland V Baird D. The MR-base platform supports systematic causal inference across the human phenome. eLife (2018) 7:e34408. doi: 10.7554/eLife.34408 29846171 PMC5976434

[16] Li B Martin EB. An approximation to the f distribution using the chi-square distribution. Comput Stat Data Anal (2002) 40(1):21–6. doi: 10.1016/s0167-9473(01)00097-4

[17] Burgess S Thompson SG CRP CHD Genetics Collaboration. Avoiding bias from weak instruments in mendelian randomization studies. Int J Epidemiol (2011) 40(3):755–64. doi: 10.1093/ije/dyr036 21414999

[18] Broadbent JR Foley CN Grant AJ Mason AM Staley JR Burgess S. MendelianRandomization v0.5.0: Updates to an r package for performing mendelian randomization analyses using summarized data. Wellcome Open Res (2020) 5:252. doi: 10.12688/wellcomeopenres.16374.2 33381656 PMC7745186

[19] Burgess S Butterworth A Thompson SG. Mendelian randomization analysis with multiple genetic variants using summarized data. Genet Epidemiol (2013) 37(7):658–65. doi: 10.1002/gepi.21758 PMC4377079 24114802

[20] Burgess S Thompson SG. Interpreting findings from mendelian randomization using the MR-egger method. Eur J Epidemiol (2017) 32(5):377–89. doi: 10.1007/s10654-017-0255-x PMC5506233 28527048

[21] Bowden J Davey Smith G Haycock PC Burgess S. Consistent estimation in mendelian randomization with some invalid instruments using a weighted median estimator. Genet Epidemiol (2016) 40(4):304–14. doi: 10.1002/gepi.21965 PMC4849733 27061298

[22] Zhang Y Liu Z Choudhury T Cornelis MC Liu W. Habitual coffee intake and risk for nonalcoholic fatty liver disease: A two-sample mendelian randomization study. Eur J Nutr (2021) 60(4):1761–7. doi: 10.1007/s00394-020-02369-z PMC7910323 32856188

[23] Carter AR Sanderson E Hammerton G Richmond RC Davey Smith G Heron J. Mendelian randomisation for mediation analysis: Current methods and challenges for implementation. Eur J Epidemiol (2021) 36(5):465–78. doi: 10.1007/s10654-021-00757-1 PMC8159796 33961203

[24] Lynch M Walsh B. Genetics and analysis of quantitative traits. Sunderland MA: Sinauer (1998) 1:535–57.

[25] Hemani G Tilling K Davey Smith G. Orienting the causal relationship between imprecisely measured traits using GWAS summary data. PloS Genet (2017) 13(11):e1007081. doi: 10.1371/journal.pgen.1007081 29149188 PMC5711033

[26] Tan JS Liu NN Guo TT Hu S Hua L. Genetically predicted obesity and risk of deep vein thrombosis. Thromb Res (2021) 207:16–24. doi: 10.1016/j.thromres.2021.08.026 34507265

[27] Tan JS Ren JM Fan L Wei Y Hu S Zhu SS. Genetic predisposition of anti-cytomegalovirus immunoglobulin G levels and the risk of 9 cardiovascular diseases. Front Cell Infect Microbiol (2022) 12:884298. doi: 10.3389/fcimb.2022.884298 35832381 PMC9272786

[28] Verbanck M Chen CY Neale B Do R. Detection of widespread horizontal pleiotropy in causal relationships inferred from mendelian randomization between complex traits and diseases. Nat Genet (2018) 50(5):693–8. doi: 10.1038/s41588-018-0099-7 PMC6083837 29686387

[29] Ferrante A Ciccia F Giammalva GR Iacopino DG Visocchi M Macaluso F. The craniovertebral junction in rheumatoid arthritis: State of the art. Acta Neurochir Suppl (2019) 125:79–86. doi: 10.1007/978-3-319-62515-7_12 30610306

[30] Shlobin NA Dahdaleh NS. Cervical spine manifestations of rheumatoid arthritis: A review. Neurosurg Rev. (2021) 44(4):1957–65. doi: 10.1007/s10143-020-01412-1 33037539

[31] Yurube T Sumi M Nishida K Miyamoto H Kohyama K Matsubara T. Incidence and aggravation of cervical spine instabilities in rheumatoid arthritis: A prospective minimum 5-year follow-up study of patients initially without cervical involvement. Spine (2012) 37(26):2136–44. doi: 10.1097/BRS.0b013e31826def1c 22895480

[32] Kotecki M Gasik R Głuszko P Sudoł-Szopińska I. Radiological evaluation of cervical spine involvement in rheumatoid arthritis: A cross-sectional retrospective study. J Clin Med (2021) 10(19):4587. doi: 10.3390/jcm10194587 34640605 PMC8509796

[33] Paus AC Steen H Røislien J Mowinckel P Teigland J. High mortality rate in rheumatoid arthritis with subluxation of the cervical spine: A cohort study of operated and nonoperated patients. Spine (2008) 33(21):2278–83. doi: 10.1097/BRS.0b013e31817f1a17 18784629

[34] Kurowska-Stolarska M Alivernini S. Synovial tissue macrophages in joint homeostasis, rheumatoid arthritis and disease remission. Nat Rev Rheumatol (2022) 18(7):384–97. doi: 10.1038/s41584-022-00790-8 35672464

[35] Sorimachi Y Iizuka H Ara T Nishinome M Iizuka Y Nakajima T. Atlanto-axial joint of atlanto-axial subluxation patients due to rheumatoid arthritis before and after surgery: Morphological evaluation using CT reconstruction. Eur Spine J (2011) 20(5):798–803. doi: 10.1007/s00586-010-1611-7 21038107 PMC3082676

[36] Nazarinia M Jalli R Kamali Sarvestani E Farahangiz S Ataollahi M. Asymptomatic atlantoaxial subluxation in rheumatoid arthritis. Acta Med Iran. (2014), 462–6. 25130155

[37] Liao S Jung MK Hörnig L Grützner PA Kreinest M. Injuries of the upper cervical spine-how can instability be identified? Int Orthop (2020) 44(7):1239–53. doi: 10.1007/s00264-020-04593-y 32451654

[38] Lv JM Chen JY Liu ZP Yao ZY Wu YX Tong CS. Celluar folding determinants and conformational plasticity of native c-reactive protein. Front Immunol (2020) 11:583. doi: 10.3389/fimmu.2020.00583 32296446 PMC7137756

[39] Fang Z Lv J Wang J Qin Q He J Wang M. C-reactive protein promotes the activation of fibroblast-like synoviocytes from patients with rheumatoid arthritis. Front Immunol (2020) 11:958. doi: 10.3389/fimmu.2020.00958 32508836 PMC7251027

[40] Nasonov EL Chichasova NV Baranov AA Imametdinova GR Tishchenko VA Kuznetsova TB. The clinical role of c-reactive protein in rheumatoid arthritis. Klin Med (Mosk) (1997) 75(7):26–30. 9411048

[41] Kim KW Kim BM Moon HW Lee SH Kim HR. Role of c-reactive protein in osteoclastogenesis in rheumatoid arthritis. Arthritis Res Ther (2015) 17(1):41. doi: 10.1186/s13075-015-0563-z 25889630 PMC4372175

[42] Möller B Everts-Graber J Florentinus S Li Y Kupper H Finckh A. Low hemoglobin and radiographic damage progression in early rheumatoid arthritis: Secondary analysis from a phase III trial. Arthritis Care Res (Hoboken) (2018) 70(6):861–8. doi: 10.1002/acr.23427 28950430

[43] Tan JS Yan XX Wu Y Gao X Xu XQ Jiang X. Rare variants in MTHFR predispose to occurrence and recurrence of pulmonary embolism. Int J Cardiol (2021) 2021:331:236–242. doi: 10.1016/j.ijcard.2021.01.073 33571559

