# Corrigendum: Genetically predicted C-reactive protein mediates the association between rheumatoid arthritis and atlantoaxial subluxation

**DOI:** 10.3389/fendo.2023.1154170

**Published:** 2023-02-23

**Authors:** Jiaqin Yuan, Xiaoqin Xiong, Bin Zhang, Qingyuan Feng, Jinglin Zhang, Wenting Wang, Jia Tang

**Affiliations:** ^1^ Department of Orthopedics, The Second People’s Hospital of Yibin, Yibin, Sichuan, China; ^2^ Department of Orthopedics, Yibin Hospital, West China Hospital of Sichuan University, Yibin, Sichuan, China; ^3^ Department of Pediatrics, The Affiliated Hospital of Southwest Medical University, Luzhou, China; ^4^ Rheumatism Immunity Branch, Weifang People’s Hospital, Weifang, Shandong, China; ^5^ Wuxi School of Medicine, Jiangnan University, Wuxi, Jiangsu, China; ^6^ Department of Occupational Disease, Yibin Center for Disease Control and Prevention, Yibin, Sichuan, China; ^7^ Department of Anesthesiology, The Second Affiliated Hospital of Hainan Medical University, Haikou, China; ^8^ Department of Pediatrics, Daping Hospital, Army Medical University, Chongqing, China

**Keywords:** Mendelian randomization, rheumatoid arthritis, C-reactive protein, atlantoaxial subluxation, upper cervical instability

**Incorrect Author Name**

In the published article, an author name was incorrectly written as Wengting Wang.

The correct spelling is Wenting Wang.

The authors apologize for this error and state that this does not change the scientific conclusions of the article in any way. The original article has been updated.

